# Pre-Frailty Phenotype and Arterial Stiffness in Older Adults Free of Cardiovascular Diseases

**DOI:** 10.3390/ijerph192013469

**Published:** 2022-10-18

**Authors:** Geovani Araújo Dantas Macêdo, Yuri Alberto Freire, Rodrigo Alberto Vieira Browne, Marcyo Câmara, Ludmila Lucena Pereira Cabral, Daniel Schwade, Ronildo Paulo-Pereira, Raíssa de Melo Silva, Alana Monteiro Bispo Silva, Luiz Fernando Farias-Junior, Todd A. Duhamel, Eduardo Caldas Costa

**Affiliations:** 1 ExCE Research Group, Department of Physical Education, Federal University of Rio Grande do Norte, Natal 59078-970, RN, Brazil; 2 Graduate Program in Health Sciences, Federal University of Rio Grande do Norte, Natal 59012-570, RN, Brazil; 3 Faculty of Kinesiology and Recreation Management, University of Manitoba, Winnipeg, MB R3T 2N2, Canada; 4 Institute of Cardiovascular Sciences, St. Boniface General Hospital Research Centre, Winnipeg, MB R3T 2N2, Canada; 5 Graduate Program in Psychobiology, Federal University of Rio Grande do Norte, Natal 59078-970, RN, Brazil

**Keywords:** frailty, cardiovascular disease, pulse wave velocity, vascular disease, aging

## Abstract

Purpose: Arterial stiffness is a subclinical marker of cardiovascular disease (CVD). The pre-frailty phenotype is associated with a higher risk for CVD. This study investigated the association between the pre-frailty phenotype and arterial stiffness in community-dwelling older adults without diagnosed CVD. Methods: In total, 249 community-dwelling older adults aged 60–80 years were included in this cross-sectional study. The pre-frailty phenotype was defined by the standardized Fried criteria (muscle weakness; slow walking speed; low physical activity; unintentional weight loss; self-reported exhaustion). Participants with one or two standardized Fried criteria were classified as pre-frail and those with zero criteria as robust. Arterial stiffness was measured by aortic pulse wave velocity (aPWV). The data were analyzed using the generalized linear model. Results: From 249 participants (66.1 ± 5.3 years; 79.5% females), 61.8% (*n* = 154) were pre-frail and 38.2% (*n* = 95) robust. Pre-frail older adults had a higher aPWV (β = 0.19 m/s; *p* = 0.007) compared to their robust peers. Conclusions: The pre-frailty phenotype was associated with higher arterial stiffness in community-dwelling older adults aged 60–80 years. Pre-frail older adults may have a higher risk for CVD.

## 1. Introduction

The risk for and mortality by cardiovascular diseases (CVD) increases with age in both males and females [1]. The prevalence of CVD is ~1% in adults aged 20–39 years, 18–27% in older adults aged 60–79 years, and 31–43% in oldest older adults aged 80+ years [1]. Studies have also reported that pre-frailty and frailty phenotypes are associated with a higher risk for and mortality by CVD in older adults [2,3]. Although frailty is a syndrome characterized by cumulative decline in multiple body systems or functions [4,5], pre-frailty and frailty phenotypes primarily focus on function [6]. First described by Fried et al. [6], the pre-frailty and frailty phenotypes are characterized by the presence of one to two– and over three of five aspects, respectively: muscle weakness, slow walking speed, low physical activity, unintentional weight loss, and self-reported exhaustion.

The pre-frailty phenotype is highly prevalent in older adults [7,8,9]. For example, a meta-analysis showed that 53% of Brazilian community-dwelling older adults are classified as pre-frail [7]. A high prevalence of pre-frailty is associated with a 4-fold higher risk for frailty [6]. Pre-frailty is defined as a complex multifactorial and multidimensional state associated with physiological and deleterious processes that developed over time [10]. Despite this, pre-frailty is clinically reversible [6,11], which highlights the importance of its early identification, as well as interventions in order to reversing it or preventing its progression to frailty.

Boreskie et al. [2] observed that pre-frail and frail females aged 55+ years have a higher risk for CVD determined by the Framingham Risk Score (pre-frail: odds ratio (OR) 1.52; frail: OR 2.81), Rasmussen Disease Score (pre-frail: OR 1.60; frail: OR 2.05), and CANHEART health index (pre-frail: OR 3.07; frail: OR 8.27) compared to robust females. In a meta-analysis conducted by Veronese et al. [3], it was observed that both pre-frail (hazard ratio (HR) 2.80; CI 95% 1.83–4.28) and frail (HR 3.89; CI 95% 2.39–6.34) older adults had a higher risk for mortality by CVD compared to robust older adults. More recently, some studies have confirmed the association of pre-frailty and frailty phenotypes with increased risk of CVD [12,13,14,15] and of developing major adverse cardiovascular events [12]. A recent meta-analysis that included 185,229 middle-aged and older adults found a 2-fold increase in risk in the frail group and 1.5-fold in the pre-frail group for mortality from CVD and cancer [13]. Therefore, investigations on early, subclinical, and non-invasive markers of CVD development in pre-frailty and frailty phenotypes can help to identify community-dwelling older adults who may be more susceptible to CVD. Given that the relationship between frailty and CVD seems to be bidirectional [3,16,17], investigating pre-frail older adults without CVD seems to be appropriate to clarify if these individuals have an increased risk for CVD indicated by early, subclinical, and non-invasive markers.

Arterial stiffness is an early and subclinical marker of CVD development which can be non-invasively assessed by pulse wave velocity (PWV) [18,19,20]. PWV is an independent predictor of CVD [21]. Orkaby et al. [9] and Nadruz et al. [8] both reported that an association between frailty phenotype and higher arterial stiffness exists. However, Orkaby et al. [9] observed an association between pre-frailty phenotype and arterial stiffness, while Nadruz et al. [8] reported that pre-frailty phenotype was not associated with arterial stiffness. It should be noted that both studies included community-dwelling older adults with coronary heart disease, peripheral artery disease, and heart failure, which precludes determining if the pre-frailty phenotype in the absence of CVD is associated with higher arterial stiffness. Thus, further investigations on the relationship between the pre-frailty phenotype and arterial stiffness are needed in older adults without known CVD. Therefore, this study investigated the association between the pre-frailty phenotype and arterial stiffness in community-dwelling older adults without diagnosed CVD.

## 2. Methods

### 2.1. Study Design

This is a cross-sectional study reported in accordance with the Strengthening the Reporting of Observational Studies in Epidemiology (STROBE) statement [22]. In addition, the data are reported as recommended by the Sex and Gender Equity in Research (SAGER) guidelines [23]. The research was conducted at the Onofre Lopes University Hospital and at the Department of Physical Education of the Federal University of Rio Grande do Norte between June 2018 and December 2019. The study was approved by the Research Ethics Board of the Onofre Lopes University Hospital (protocol 2.603.422/2018) and conducted in accordance with the Declaration of Helsinki. All participants were informed about the study procedures and gave written informed consent.

### 2.2. Participants

**Figure 1:**
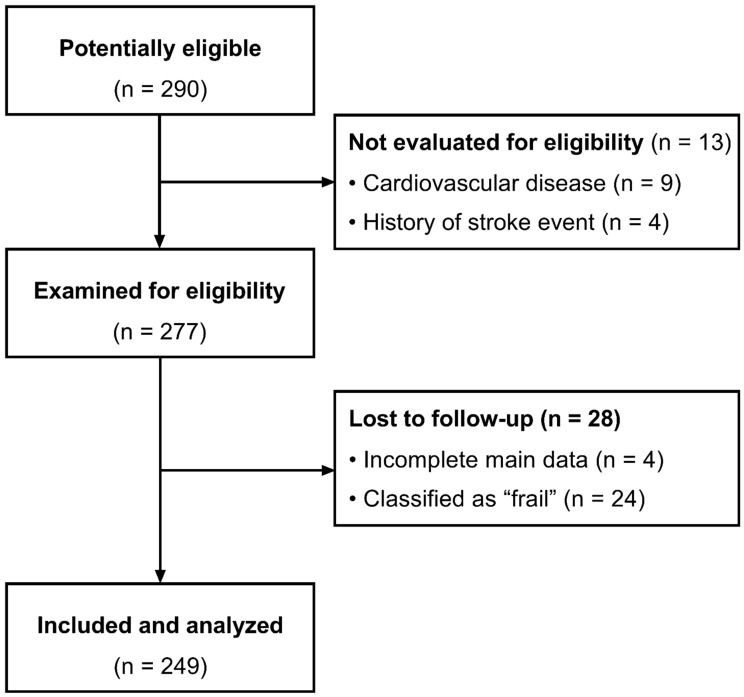
Study flowchart.

A sample of community-dwelling Brazilian older adults was selected for this study. Participants were selected in the city of Natal, capital of the state of Rio Grande do Norte, Brazil through radio, TV, e-flyers in social media, healthcare units, and older adult community centers. A total of 290 participants were screened for this study. Among these participants, 277 participants were eligible. Eligibility criteria were as follows: (i) no history of known cardiovascular diseases or major cardiovascular events (e.g., acute myocardial infarction, stroke, coronary artery disease, arrhythmias, or peripheral vascular disease), (ii) no joint or musculoskeletal injury that limits the ability to perform exercise, and (iii) no acute diabetes- or hypertension-related decompensation (i.e., glycemia ≥300 mg/dL; blood pressure ≥160/105 mmHg). Participants classified as ‘frail’ (i.e., 3+ standardized Fried or original Fried criteria) or with incomplete data were excluded from the final analysis. Figure 1 shows the study flowchart.

### 2.3. Fried Frailty Phenotypes

Previous studies [2,24,25] have highlighted the importance of using cohort-specific cut-offs to define frailty phenotype criteria, mainly for cohorts that are younger than those from the original Fried study, which included older adults aged 65–101 years. Furthermore, Boreskie et al. [2] demonstrated that the Fried frailty phenotype better discriminates CVD risk when standardized to the cohort population. Thus, the standardized Fried frailty phenotype was used for data analyses as the present study included older adults aged 60–80 years. All data analyses were then repeated using the original Fried frailty phenotype cut-offs and the data are included as Supplementary Files.

The pre-frailty phenotype is characterized as the presence of one or two of five frailty markers: low physical activity, weakness (low grip strength), slowness (slow walking speed), self-reported exhaustion, and unintentional weight loss. The participants with no criteria were classified as robust [6]. For the standardized Fried frailty phenotype, new standardized cut-offs for low grip strength, slow walking speed, and low physical activity were determined according the recommendations of Bouzón et al. [25]. Low grip strength cut-offs were defined as the lowest quintile according to sex and BMI of this specific study cohort. Slow walking speed cut-offs were defined as the lowest cohort-specific quintile according to sex and height. Low physical activity cut-offs were defined as the lowest quintile according to sex for this specific cohort. Physical activity level was assessed by the Minnesota Leisure Activity Questionnaire [26]. The physical activities were classified according to their metabolic equivalents (METs), based on the compendium of physical activity [27]. Self-reported exhaustion and unintentional weight loss cut-offs were defined using the same criteria as the original Fried frailty phenotype [6]. Self-reported exhaustion was assessed by the Center for Epidemiological Studies Depression Scale [28]. The following two statements were read: (i) “I felt that everything I did was an effort”; (ii) “I could not get going”. The participants who reported the frequency of 3+ days per week for the above-mentioned statements reached the exhaustion criterion [6]. Unintentional weight loss was defined as a loss of more than 10 pounds or 5% of body weight in the last 12 months not associated with diet or exercise [6].

### 2.4. Arteral Stiffnesis and Blood Pressure

The aortic pulse wave velocity (aPWV) and central and brachial blood pressure (BP) values were measure using the non-invasive automatic oscillometric device that detects oscillometric waves of brachial BP (Dyna-Mapa AOP^®^, Cardios, São Paulo, Brazil). The device uses a mathematical model considering parameters from pulse wave analysis and wave separation analysis by ARCSolver^®^ algorithms (Austrian Institute of Technology, Vienna, Austria) to estimate aPWV [29,30].

The algorithm was used to obtain conventional blood pressure. The brachial cuff is inflated to record the pulse waves. The central pressure curves obtained through a transfer function from the peripheral reading are plotted. To estimate the aPWV, the ARCSolver method utilizes several parameters from pulse wave analysis and wave separation analysis combined in a proprietary mathematical model. The aPWV obtained from this non-invasive device was validated by comparing it with the aPWV obtained from the gold standard, the invasive intra-aortic catheter method (the mean difference = 0.43 m/s ± 1.24 m/s; correlation: R = 0.81, *p* < 0.0001; 95%CI 0.74 to 0.86) [31,32], and from the non-invasive applanation tonometry (the mean difference ~0.5 mm Hg ± 4.7 mm Hg) [33,34,35]. The aPWV obtained from this non-invasive device showed the mean difference in repeated measurements was 0.05 m/s, and the coefficient of variation was 2.14% [31]. Moreover, in the algorithm validation study (ARCSolver), appropriate results were found compared with SphygmoCor (mean difference = −0.1 ± 3.1 mmHg), showing good compliance with SphygmoCor in different age groups, including the older adult population [36]. The aPWV and central and brachial BP values were assessed following the manufacturer’s instructions. Four consecutive measures were obtained in the left arm with a 1-min interval between each one. The participant remained in a supine position during measures in a quiet and temperature-controlled room (24–26 °C). The mean value of aPWV (m/s) and central and brachial BP values obtained from the three measures were considered for data analysis. In addition, the following variables were considered: (i) central systolic BP (cSBP), (ii) central diastolic BP (cDBP), (iii) central mean BP (cMBP), (iv) central pulse pressure (cPP), (vi) brachial systolic BP (bSBP), (vii) brachial diastolic BP (bDBP), (viii) brachial mean BP (bMBP), and (ix) brachial pulse pressure (bPP).

### 2.5. Blood Parameters and Framingham Risk Score

Blood samples were collected following a 12-h overnight fasting period by venipuncture in order to assess total cholesterol, HDL-cholesterol, LDL-cholesterol, triglycerides, and fasting glucose. LDL-cholesterol was determined using the Friedewald formula: (total cholesterol—[HDL + triglycerides/5]). All biochemical assays were determined using commercial kits (Diagnostic Labtest-SA^®^, São Paulo, Brazil) by the colorimetric method (Labtest^®^, Labmax Plenno, Minas Gerais, Brazil). The Framingham risk score for CVD was calculated using the following criteria: age, sex, smoking, BMI, systolic BP, and medication use for hypertension and diabetes [37]. Cut-offs of <10%, 10–19%, and ≥20% were used to determine low, moderate, and high risk, respectively.

### 2.6. Other Variables

Additional information was obtained from the participants by a face-to-face interview: age, education, smoking history, medication use for hypertension, diabetes, and dyslipidemia. Body weight (kg) and height (m) were measured, and BMI was calculated as weight (kg) divided by the square of height in meters (kg/m^2^).

### 2.7. Statistical Analysis

Descriptive data are expressed as mean and standard deviation or as absolute and relative frequencies. One-way ANOVA with multiple comparisons by the LSD test was used to compare the continuous data between pre-frailty and robust groups. Fisher’s exact test was used to compare the categorical data between the pre-frailty and robust groups. Age- and fully adjusted generalized linear models (GzLM) assessed the associations between aPWV and pre-frailty phenotype (robust as reference). The maximum likelihood estimation was used to calculate the parameter estimates (β) and 95% Wald confidence intervals (CI). The multiple models were adjusted for variables that were associated with aPWV in the bivariate analyses at *p*-value < 0.20 and in the multivariate model at *p*-value < 0.10 [38]. The following variables were considered as potential confounders: age, sex, education, partner, BMI, fasting glucose, triglycerides, HDL-cholesterol, LDL-cholesterol, total cholesterol, resting BP, lipid-lowering medication(s), diabetes-lowering medication(s), antihypertensive medication(s), and smoking status. The linear or gamma distribution model for each model was defined by the normality of the residuals in the normal Q-Q plot and/or by the lower value of Akaike Information Criterion (AIC). An omnibus test was performed to assess the goodness-of-fit for the models. Statistical significance was set at 5% for all analyses, which were performed using IBM SPSS Statistics^®^ for Win/v.25.0 (IBM Corp., Armonk, NY, USA).

## 3. Results

Table 1 shows the characteristics of the 249 participants included in the final analysis using the standardized Fried criteria. Most participants were females (79.5%, *n* = 198), living with a partner (63.9%, *n* = 159), and had diagnosed hypertension (61.4%, *n* = 153) and excess body weight (overweight: 20.1%, *n* = 50; obesity: 43.4%, *n* = 108). Approximately half of participants were smokers/ex-smokers (44.2%, *n* = 110) and had moderate CVD risk determined by the Framingham risk score (49.2%, *n* = 121). The prevalence of pre-frailty phenotype was 61.8% (*n* = 154). A total of 39.0% (*n* = 97) and 22.9% (*n* = 57) of participants met one or two of the standardized Fried criteria for the pre-frailty phenotype, respectively. Pre-frail participants had higher cSBP and cPP, bSBP, and bPP (*p* < 0.05) compared to the robust participants. Finally, the most prevalent standardized Fried frailty criterion was low physical inactivity, followed by exhaustion, weakness, slowness, and unintentional weight loss. Table S1 shows the characteristics of the participants using the original Fried criteria. Tables S4 and S5 show the characteristics of the participants according to sex using both standardized and original Fried criteria. Using the standardized Fried criteria, females had a higher prevalence of living with partner (51.6%, *n* = 64), while males had a higher prevalence of post-secondary education (33.3%, *n* = 10) and lipid medication use (33.9%, *n* = 42). No differences were observed regarding the characteristics of included and excluded participants.

**Figure 2:**
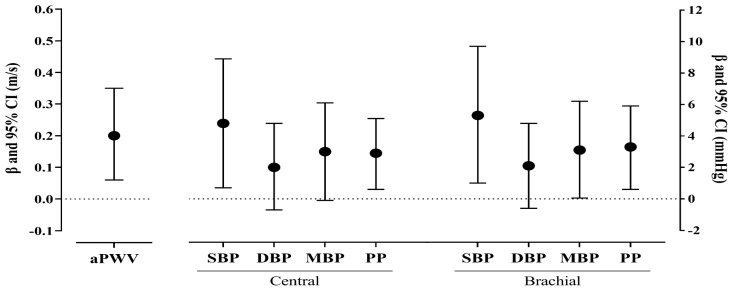
Aortic pulse wave velocity and blood pressure (central and brachial) in pre-frail vs. robust older adults based on standardized Fried criteria. Notes: Values are shown as coefficient estimates (β) and 95% confidence interval (CI). Adjusted model for age, sex, body mass index categories, post-secondary education, and hypertension medication (robust as reference). Abbreviations: aPWV, aortic pulse wave velocity; BP, blood pressure; SBP, systolic blood pressure; DBP, diastolic blood pressure; MBP, mean blood pressure; PP, pulse pressure. Figure S1 and Table S3 show the results of GzLM for aPWV, cBP, and bBP based on the original Fried criteria. In the fully adjusted GzLM, pre-frail participants showed an aPWV 0.19 m/s higher compared to the robust participants (*p* = 0.010). In addition, pre-frail older adults showed a higher cSBP (β = 4.7 mmHg, *p* = 0.023), cMBP (β = 3.2 mmHg, *p* = 0.043), cPP (β = 2.3 mmHg, *p* = 0.045), bSBP (β = 5.8 mmHg, *p* = 0.007), bMBP (β = 3.7 mmHg, *p* = 0.018), and bPP (β = 3.2 mmHg, *p* = 0.016) compared to the robust older adults.

Figure 2 and Table S2 show the results of GzLM for aPWV, cBP, and bBP for standardized Fried criteria. In the fully adjusted GzLM, pre-frail participants showed aPWV 0.19 m/s higher compared to the robust participants (*p* = 0.007). In addition, pre-frail older adults showed a higher cSBP (β = 4.8 mmHg, *p* = 0.021), cPP (β = 2.9 mmHg, *p* = 0.014), bSBP (β = 5.3 mmHg, *p* = 0.017), bMBP (β = 3.1 mmHg, *p* = 0.046), and bPP (β = 3.3 mmHg, *p* = 0.017) compared to the robust older adults.

Furthermore, in following the SAGER guidelines [23], we included the results of GzLMs according to sex as Supplementary Tables, including both the standardized and original Fried criteria (Tables S4–S7). In the fully adjusted GzLM according to the sex for standardized Fried criteria (Table S6), pre-frail participants of both sexes showed an aPWV higher compared to the robust participants (male: aPWV 0.32 m/s, *p* = 0.023, and female: aPWV 0.18 m/s, *p* = 0.043). However, only male pre-frail older adults showed a higher cSBP (β = 9.0 mmHg, *p* = 0.050), cMBP (β = 6.4 mmHg, *p* = 0.049), cPP (β = 4.9 mmHg, *p* = 0.042), bSBP (β = 9.6 mmHg, *p* = 0.045), bMBP (β = 6.4 mmHg, *p* = 0.046), and bPP (β = 5.1 mmHg, *p* = 0.027) compared to their robust peers.

Table S7 showed the fully adjusted GzLM according to the sex for original Fried criteria. The male pre-frail participants showed a higher aPWV (0.37 m/s, *p* = 0.014), cSBP (β = 9.2 mmHg, *p* = 0.043), cPP (β = 5.7 mmHg, *p* = 0.004), bSBP (β = 9.8 mmHg, *p* = 0.044), and bPP (β = 6.3 mmHg, *p* = 0.008) compared to their robust peers. However, female pre-frail older adults only showed a higher bSBP (β = 4.9 mmHg, *p* = 0.047) compared to their robust peers.

## 4. Discussion

This study investigated the association between pre-frailty phenotype and arterial stiffness in community-dwelling older adults without diagnosed CVD. Our main findings indicate that pre-frail older adults showed higher arterial stiffness (aPWV; 0.19 m/s) compared to robust older adults. In addition, pre-frailty was associated with higher cBP and bBP. Of note, these findings were consistent using both the standardized and the original Fried frailty phenotype criteria.

The prevalence of pre-frail phenotype based on the standardized Fried criteria in our study was similar to the prevalence reported in a recent meta-analysis including non-institutionalized Brazilian older adults with mean age varying from ~65 to ~85 years (54%) [7]. In addition, the three most common Fried criteria in our study (low physical activity = 35.7%; exhaustion = 30.5%; weakness = 26.6%) were similar to those observed by Melo et al. [7], although the order and prevalence were different (exhaustion = 32%; weakness = 26%; low physical activity = 27%). Thus, the prevalence of pre-frailty phenotype and its more common components found in our study seem to be similar to observed in the Brazilian population aged 60+ years.

Little is known about the relationship between the pre-frailty phenotype and arterial stiffness in older adults to date. Two available studies have conflicting results. Data from the Framingham Heart Study [9] reported higher carotid-femoral PWV in pre-frail (10.3 m/s, 95% CI 10.2–10.5) and frail (10.5 m/s, CI 95% 10.1–11.0) community-dwelling older adults compared to robust adults (10.0 m/s, CI 95% 9.9–10.1). The association between pre-frailty and frailty phenotypes with arterial stiffness was similar in males and females and in different age groups (i.e., ≤69 vs. ≥70 years). On the other hand, data from the Atherosclerosis Risk in Communities Study [8] reported higher arterial stiffness (carotid-femoral PWV or ankle-brachial index) in community-dwelling frail but not in pre-frail older adults compared to robust adults. Both of the above-mentioned studies included older adults with different characteristics compared to our study, i.e., older participants (~70 and ~75 years), mostly Caucasian (~90 and 75%), and individuals with or without CVD [8,9]. Our findings are similar to those observed by Orkaby et al. [9], even though our sample population of community-dwelling adults aged 60–80 years are of a different ethnicity and without a history of CVD.

The aPWV is a subclinical predictor of CVD development and major cardiovascular events [39]. Pre-frail older adults showed a 0.19 m/s higher aPWV compared to robust adults, which is clinically meaningful and associated with a ~2% increase in CVD risk [39]. Although this increased risk does not necessarily impose changes in cardiovascular risk management among robust and pre-frail older adults, it warns of an urgency in cardiovascular health management in pre-frail older adults [40]. Among pre-frail older adults, ~62% and ~32% reported low physical activity and exhaustion, respectively. These frailty components are likely to contribute to unhealthy movement behavior in pre-frail older adults compared to their robust peers. The absence of moderate-to-vigorous physical activity combined with prolonged sitting time reduces the blood flow and shear stress in the vasculature, which impairs the flow-mediated dilation and contributes to endothelial dysfunction [41]. The reduction in shear stress stimulates the production of endothelin-1 (ET-1), which in turn reduces the NO bioavailability, an important signal for vasodilation [42,43]. Over time, this unhealthy movement behavior pattern is likely to contribute to endothelial damage and arterial stiffness [44]. Previous studies have reported that low moderate-to-vigorous physical activity [45] and steps per day [46], as well as high sedentary time [45], are associated with arterial stiffness, which may partially explain our findings. However, cause and effect extrapolations cannot be made due to the study design. Therefore, future studies are needed to investigate the relationship between movement/sedentary behaviors and arterial stiffness in the pre-frailty phenotype to confirm or disprove our preliminary findings. In addition to the physical aspect of physical exhaustion, Fried et al. [6] indicate this criterion as a depressed mood typical of frail individuals. Veronese et al. [47] suggest that exhaustion is the main criterion associated with incidence of CVD. Corroborating the importance of this depressed characteristic, which is part of the exhaustion criterion, a meta-analysis with 3,211,768 patients and 113,383,368 controls showed a positive association between depressive signs and risk of CVD and CVD-related mortality [48].

This study identified an association between the pre-frailty phenotype with higher cBP and bBP in older adults aged 60–80 years without diagnosed CVD. Pre-frail older adults had ~5 mmHg higher cSBP and bSBP than their robust peers. Both cSBP and bSBP are clinically meaningful predictors of major cardiovascular events and CVD mortality in middle-aged and older adults [49,50]. Recently, Lamarche et al. [51] reported that both cSBP (HR 1.16, 95% CI 1.10–1.22) and bSBP (HR 1.15, 95% CI 1.09–1.22) were associated with major cardiovascular events in older adults. Additionally, the authors [51] observed a significant 5 mmHg higher cSBP (117 ± 16 vs. 112 ± 14 mmHg) and bSBP (127 ± 17 vs. 122 ± 15 mmHg) in individuals with incidence of major cardiovascular events compared to those without events. In a prospective cohort, Zheng et al. [52] observed that an increase of 5 mmHg in bSBP was associated with a higher risk for stroke in older adults with hypertension (HR 1.36, CI 95% 1.22–1.52). Both studies [51,52] included older adults aged ~60 years, which is similar to our study. bSBP has a U-shaped association with all-cause mortality in older adults aged 80+ years, which is likely to be explained due to the association of high bSBP values (>154 mmHg) with CVD mortality and low bSBP values (<107 mmHg) with non-CVD mortality [53].

Studies have reported conflicting findings regarding the association between pre-frailty phenotype with diagnosed hypertension, use of antihypertensive medication(s), and bBP levels [2,54,55,56,57,58,59,60]. Some studies observed a higher prevalence of diagnosed hypertension and/or use of antihypertensive medication(s) in pre-frail older adults compared to their robust peers [2,54,55,60], while other studies did not observe this association [56,57,58,59]. A similar inconsistency regarding the association between pre-frailty with diagnosed hypertension, use of antihypertensive medication(s), and bBP levels has been found when frailty status was assessed by different models and tools [57,61,62,63]. It seems that the characteristics of the populations investigated, including age (< 80 years vs. 80+ years) and the presence (or not) of CVD, the BP measurement methods (office, home, and ambulatory) and the models and tools used to assess frailty status may be associated with the above-mentioned conflicting findings.

When analyzed according to sex, both pre-frail males and females had a higher aPWV compared to their robust peers. However, only males showed higher BP values in the pre-frail group compared to the robust group. We believe that despite the higher prevalence of hypertension in females, a better BP control due the use of more antihypertensive medications may explain this result. Similarly, a study that analyzed data from 3651 participants in the Lausanne cohort Lc65+ [54] showed a better BP control in females compared to males, regardless of frailty status and age. Additional studies aiming to investigate the relationship between BP and frailty status in different subgroups, with and without the use of antihypertensive medication(s), comorbidities, and other cardiovascular risk factors are needed to better understand the relationship between BP and frailty status.

This study has some limitations. It is not possible to establish a cause–effect relationship between the pre-frailty phenotype and arterial stiffness because it is a cross-sectional study. Longitudinal studies are needed to better understand this relationship. Only older adults aged 60–80 years were included. Therefore, our findings are not transferable to adults younger than 60 or older than 80 years of age. The participants were recruited by diverse advertisement methods, but we do not rule out the possibility of selection bias due to the need for transportation to the research laboratory. This might have limited the participation of older adults with poor mobility. Our study does not present data on the reproducibility of our sample regarding the aPWV data. However, we are using data from a previous study that used the same device to measure aPWV [31]. This previous study showed good measurement reproducibility in the non-invasive method (mean difference in repeated measurements was 0.05 m/s, and the coefficient of variation was 2.14%) [31]. Despite this, the difference found in our findings is continuous in the margin of error of the measurement presented by Hametner et al. [31] of CVD risk. Therefore, future studies aiming to verify the reproducibility of the measurement in the sample similar to our study, in addition to studies with more accurate methods, are necessary to better verify this difference found between the robust and pre-frail older adults.

From a clinical perspective, our findings support the idea that community-dwelling pre-frail older adults aged 60–80 years have elevated CVD risk based on higher aPWV, cBP, and bBP values compared to robust older adults. Thus, early identification of pre-frail older adults may help to identify individuals with elevated CVD risk, which may enable those individuals and their health care providers to provide clinical treatment or lifestyle recommendations so the individuals can better manage their CVD risk and avoid future adverse cardiovascular events. For example, pre-frail older adults should be encouraged to take up lifestyle interventions designed to enhance overall cardiovascular health, reduce arterial stiffness, and better manage BP. It is important to highlight that aerobic exercises promote improvements in the functional role of the arteries. Increased flow-mediated dilatation capacity, greater NO bioavailability, and lower ET-1 expression [40] are promoted by more muscle activity. The consequent increase in blood flow in the lower limbs, contributing to the reduction of arterial stiffness [64] and BP [65]. However, the effect of exercise on the structural composition of arteries in humans is still unclear [66]. The elastin and collagen, which are related to the morphology of the arteries, are components of chronic modifications in arteries. To date, there is no consistent evidence that physical exercise modifies the morphology of the arteries in humans [66]. Furthermore, a meta-analysis reported that an increase of 1000 steps per day reduces ~0.18 m/s in PWV [46]. Resistance training is also able to reduce BP [65], although its effects on arterial stiffness seem to be limited [67]. Aerobic exercise training reduces arterial stiffness [64] and BP [65].

## 5. Conclusions

The pre-frailty phenotype was associated with a higher arterial stiffness in community-dwelling older adults aged 60–80 years without CVD. As arterial stiffness is an earlier and subclinical marker of CVD development, our findings suggest that pre-frail older adults have a higher risk for CVD. Longitudinal data are needed to confirm (or not) the above-mentioned assumption.

## Figures and Tables

**Table 1:** Characteristics of the participants based on the standardized Fried criteria.

	Overall	Robust	Pre-Frail	*p*
*n* (%)	249	95 (38.2)	154 (61.8)	
Age, years	66.1 ± 5.3	66.5 ± 5.3	65.9 ± 5.4	0.392
Race, n (%)				
Caucasian	89 (35.7)	32 (33.7)	57 (37)	0.733
Brown	138 (55.4)	56 (58.9)	82 (53.2)	
Black	17 (6.8)	6 (6.3)	11 (7.1)	
Other	5 (2)	1 (1.1)	5 (2.6)	
Females, n (%)	198 (79.5)	74 (77.9)	124 (80.5)	0.618
Living with partner, n (%)	159 (63.9)	68 (71.6)	91 (59.1)	0.046
Post-secondary education, n (%)	51 (20.5)	16 (16.8)	35 (22.7)	0.264
Body mass index, kg/m^2^	29 ± 4.6	28.6 ± 4.3	29.2 ± 4.8	0.314
Fasting glucose, mg/dL	111.1 ± 31.9	111.7 ± 36.8	110.8 ± 28.6	0.836
Triglycerides, md/dL	154.3 ± 75	157.9 ± 98.9	152.1 ± 64.2	0.552
HDL-cholesterol, md/dL	45.9 ± 12.7	45.3 ± 11.6	46.3 ± 13.3	0.556
LDL-cholesterol, md/dL	132.4 ± 44.6	135 ± 47.4	130.8 ± 42.9	0.473
Total cholesterol, md/dL	205.2 ± 46.7	205.9 ± 48.1	204.7 ± 46	0.838
Antihypertensive medication, n (%)				
Monotherapy	73 (48)	30 (50.8)	43 (46.2)	0.579
Combination therapy	79 (52)	29 (49.2)	50 (53.8)	0.579
Calcium channel blockers	16 (10.5)	6 (10.2)	10 (10.8)	0.909
Diuretics	61 (40.1)	19 (32.2)	42 (45.2)	0.112
Angiotensin II receptor blockers	116 (76.3)	45 (76.3)	71 (76.3)	0.992
ACE inhibitors	14 (9.2)	5 (8.5)	9 (9.7)	0.803
Beta-blockers	39 (25.7)	20 (33.9)	19 (20.4)	0.064
Diabetes medication, n (%)	64 (25.7)	29 (30.5)	35 (22.7)	0.171
Lipid medication, n (%)	80 (32.1)	24 (25.3)	56 (36.4)	0.068
Ex-smoker/smoker, n (%)	110 (44.2)	40 (42.1)	70 (45.5)	0.605
Framingham risk, n (%)				
Low risk	82 (33.3)	36 (38.3)	46 (30.3)	0.347
Moderate risk	121 (49.2)	41 (43.6)	80 (52.6)	
High risk	43 (17.5)	17 (18.1)	26 (17.1)	
Central SBP, mmHg	121 ± 16.4	118.1 ± 15.8	122.7 ± 16.5	0.029
Central DBP, mmHg	82.1 ± 11.4	80.9 ± 9.6	82.9 ± 12.3	0.174
Central MBP, mmHg	95.1 ± 12.5	93.3 ± 11.1	96.2 ± 13.1	0.075
Central PP, mmHg	38.8 ± 9.5	37.2 ± 9.7	39.8 ± 9.2	0.032
Brachial SBP, mmHg	128.4 ± 17.4	125.3 ± 16.7	130.3 ± 17.6	0.027
Brachial DBP, mmHg	81 ± 11.1	79.7 ± 9.4	81.8 ± 12	0.147
Brachial MBP, mmHg	96.8 ± 12.5	94.9 ± 11.1	98 ± 13.2	0.058
Brachial PP, mmHg	47.3 ± 11	45.5 ± 11.5	48.5 ± 10.6	0.043
Aortic pulse wave velocity, m/s	9.6 ± 1.1	9.5 ± 1.1	9.6 ± 1.1	0.544
Standardized Fried frailty, n (%)				
Low physical activity	-	-	55 (35.7)	
Exhaustion	-	-	47 (30.5)	
Weakness	-	-	41 (26.6)	
Unintentional weight loss	-	-	29 (18.8)	
Slowness	-	-	39 (25.3)	

**Notes:** Values are shown as mean ± SD or absolute (*n*) and relative (%) frequency. Bold values indicate statistical significance between pre-frail and robust. (*p* < 0.05). **Abbreviations:** ACE, angiotensin-converting-enzyme; BP, blood pressure; DBP, diastolic blood pressure; HDL, high-density lipoprotein; LDL, low-density lipoprotein; SBP, systolic blood pressure; MBP, mean blood pressure; PP, pulse pressure.

## Data Availability

https://osf.io/uph2b/?view_only=7207cf9f3ff84d6bb43cb470810eadca (accessed on 10 October 2022).

## Supplementary Materials

The following supporting information can be downloaded at: https://www.mdpi.com/article/10.3390/ijerph192013469/s1, Table S1: Characteristics of the participants based on the original Fried criteria; Table S2: Coefficient estimates for aortic pulse wave velocity and blood pressure (central and brachial) among pre-frail vs. robust older adults based on the standardized Fried criteria; Table S3: Coefficient estimates for aortic pulse wave velocity and blood pressure (central and brachial) among pre-frail vs. robust older adults based on the original Fried criteria; Table S4: Characteristics of the participants based on the standardized Fried criteria according to sex; Table S5: Characteristics of the participants based on the original Fried criteria according to sex; Table S6: Coefficient estimates for aortic pulse wave velocity and blood pressure (central and brachial) among pre-frail vs. robust older adults based on the standardized Fried criteria according to sex; Table S7: Coefficient estimates for aortic pulse wave velocity and blood pressure (central and brachial) among pre-frail vs. robust older adults based on the original Fried criteria according to sex; Figure S1: Aortic pulse wave velocity and blood pressure (central and brachial) in pre-frail vs. robust older adults for original Fried criteria (*n* = 259).

## References

[1] Virani S.S., Alonso A., Aparicio H.J., Benjamin E.J., Bittencourt M.S., Callaway C.W., Carson A.P., Chamberlain A.M., Cheng S., Delling F.N. (2021). Heart Disease and Stroke Statistics—2021 Update: A Report from the American Heart Association. Circulation.

[2] Boreskie K.F., Rose A.V., Hay J.L., Kehler D.S., Costa E.C., Moffatt T.L., Arora R.C., Duhamel T.A. (2020). Frailty status and cardiovascular disease risk profile in middle-aged and older females. Exp. Gerontol..

[3] Veronese N., Cereda E., Stubbs B., Solmi M., Luchini C., Manzato E., Sergi G., Manu P., Harris T., Fontana L. (2017). Risk of cardiovascular disease morbidity and mortality in frail and pre-frail older adults: Results from a meta-analysis and exploratory meta-regression analysis. Ageing Res. Rev..

[4] Clegg A., Young J., Iliffe S., Rikkert M.O., Rockwood K. (2013). Frailty in elderly people. Lancet.

[5] Morley J.E., Vellas B., van Kan G.A., Anker S.D., Bauer J.M., Bernabei R., Cesari M., Chumlea W., Doehner W., Evans J. (2013). Frailty Consensus: A Call to Action. J. Am. Med. Dir. Assoc..

[6] Fried L.P., Tangen C.M., Walston J., Newman A.B., Hirsch C., Gottdiener J., Seeman T., Tracy R., Kop W.J., Burke G. (2001). Frailty in Older Adults: Evidence for a Phenotype. J. Gerontol. Ser. A Biol. Sci. Med. Sci..

[7] Melo R.C., Cipolli G.C., Buarque G.L.A., Yassuda M.S., Cesari M., Voshaar R.C.O., Aprahamian I. (2020). Prevalence of Frailty in Brazilian Older Adults: A Systematic Review and Meta-Analysis. J. Nutr. Health Aging.

[8] Nadruz W., Kitzman D., Windham B.G., Kucharska-Newton A., Butler K., Palta P., Griswold M.E., Wagenknecht L.E., Heiss G., Solomon S.D. (2017). Cardiovascular Dysfunction and Frailty Among Older Adults in the Community: The ARIC Study. J. Gerontol. Ser. A.

[9] Orkaby A.R., Lunetta K., Sun F.J., Driver J.A., Benjamin E.J., Hamburg N., Mitchell G.F., Vasan R.S., Murabito J.M. (2019). Cross-Sectional Association of Frailty and Arterial Stiffness in Community-Dwelling Older Adults: The Framingham Heart Study. J. Gerontol. Ser. A.

[10] Sezgin D., Liew A., O’Donovan M.R., O’Caoimh R. (2020). Pre-frailty as a multi-dimensional construct: A systematic review of definitions in the scientific literature. Geriatr. Nurs..

[11] Ofori-Asenso R., Chin K.L., Mazidi M., Zomer E., Ilomaki J., Ademi Z., Bell J.S., Liew D. (2019). Natural Regression of Frailty Among Community-Dwelling Older Adults: A Systematic Review and Meta-Analysis. Gerontologist.

[12] Damluji A.A., Chung S.E., Xue Q.L., Hasan R.K., Moscucci M., Forman D.E., Bandeen-Roche K., Batchelor W., Walstom J.D., Gerstenblith G. (2021). Frailty and cardiovascular outcomes in the National Health and Aging Trends Study. Eur. Heart J..

[13] Peng Y., Zhong G.C., Zhou X., Guan L., Zhou L. (2022). Frailty and risks of all-cause and cause-specific death in community-dwelling adults: A systematic review and meta-analysis. BMC Geriatr..

[14] Liu X., Dai G., He Q., Ma H., Hu H. (2022). Frailty Index and Cardiovascular Disease among Middle-Aged and Older Chinese Adults: A Nationally Representative Cross-Sectional and Follow-Up Study. J. Cardiovasc. Dev. Dis..

[15] Hou Y., Xu C., Lu Q., Zhang Y., Cao Z., Li S., Yang H., Sun L., Cao X., Zhao Y. (2022). Associations of frailty with cardiovascular disease and life expectancy: A prospective cohort study. Arch. Gerontol. Geriatr..

[16] Afilalo J., Alexander K.P., Mack M.J., Maurer M.S., Green P., Allen L.A., Popma J.J., Ferrucci L., Forman D.E. (2014). Frailty Assessment in the Cardiovascular Care of Older Adults. J. Am. Coll. Cardiol..

[17] Frisoli A., Ingham S.J.M., Paes T., Tinoco E., Greco A., Zanata N., Pintarelli V., Elber I., Borges J., Carvalho A.C.C. (2015). Frailty predictors and outcomes among older patients with cardiovascular disease: Data from Fragicor. Arch. Gerontol. Geriatr..

[18] Kaess B.M., Rong J., Larson M.G., Hamburg N.M., Vita J.A., Levy D., Benjamin E.J., Vasan R.S., Mitchell G.F. (2012). Aortic Stiffness, Blood Pressure Progression, and Incident Hypertension. JAMA.

[19] Mitchell G.F., Hwang S.-J., Vasan R.S., Larson M., Pencina M.J., Hamburg N., Vita J., Levy D., Benjamin E. (2010). Arterial Stiffness and Cardiovascular Events. Circulation.

[20] Mitchell G.F., Parise H., Benjamin E.J., Larson M.G., Keyes M.J., Vita J.A., Vasan R.S., Levy D. (2004). Changes in Arterial Stiffness and Wave Reflection with Advancing Age in Healthy Men and Women. Hypertension.

[21] Kim H.-L., Kim S.-H. (2019). Pulse Wave Velocity in Atherosclerosis. Front. Cardiovasc. Med..

[22] Von Elm E., Altman D.G., Egger M., Pocock S.J., Gøtzsche P.C., Vandenbroucke J.P., Strobe Initiative Faculty (2014). Opinions recommendation of The Strengthening the Reporting of Observational Studies in Epidemiology (STROBE) statement: Guidelines for reporting observational studies. Int. J. Surg..

[23] Heidari S., Babor T.F., De Castro P., Tort S., Curno M. (2016). Sex and Gender Equity in Research: Rationale for the SAGER guidelines and recommended use. Res. Integr. Peer Rev..

[24] Boreskie K.F., Kehler D.S., Costa E.C., Hiebert B.M., Hamm N.C., Moffatt T.L., Hay J.L., Stammers A.N., Kimber D.E., Kent D.E. (2019). Standardization of the Fried frailty phenotype improves cardiovascular disease risk discrimination. Exp. Gerontol..

[25] Bouzón C.A., Carnicero J.A., Turín J.G., García-García F.J., Esteban A., Rodríguez-Mañas L. (2017). The Standardization of Frailty Phenotype Criteria Improves Its Predictive Ability: The Toledo Study for Healthy Aging. J. Am. Med. Dir. Assoc..

[26] Lustosa L.P., Pereira D.S., Dias R.C., Britto R.R., Parentoni A.N., Pereira L.S.M. (2011). Tradução e adaptação transcultural do Minnesota Leisure Time Activities Questionnaire em idosos. Geriatr. Gerontol..

[27] Ainsworth B.E., Haskell W.L., Herrmann S.D., Meckes N., Bassett D.R., Tudor-Locke C., Greer J.L., Vezina J., Whitt-Glover M.C., Leon A.S. (2011). 2011 Compendium of Physical Activities: A Second Update of Codes and MET Values. Med. Sci. Sport. Exerc..

[28] Radloff L.S. (1977). The CES-D Scale: A Self-Report Depression Scale for Research in the General Population. Appl. Psychol. Meas..

[29] The Reference Values for Arterial Stiffness’ Collaboration (2010). Determinants of pulse wave velocity in healthy people and in the presence of cardiovascular risk factors: ‘establishing normal and reference values’. Eur. Heart J..

[30] Wassertheurer S., Mayer C.C., Breitenecker F. (2008). Modeling arterial and left ventricular coupling for non-invasive measurements. Simul. Model. Pract. Theory.

[31] Hametner B., Wassertheurer S., Kropf J., Mayer C.C., Eber B., Weber T. (2013). Oscillometric estimation of aortic pulse wave velocity. Blood Press. Monit..

[32] Papaioannou T.G., Karageorgopoulou T.D., Sergentanis T.N., Protogerou A., Psaltopoulou T., Sharman J., Weber T., Blacher J., Daskalopoulou S.S., Wassertheurer S. (2016). Accuracy of commercial devices and methods for noninvasive estimation of aortic systolic blood pressure a systematic review and meta-analysis of invasive validation studies. J. Hypertens..

[33] Hoshide S., Komori T., Ogata Y., Eguchi K., Kario K. (2018). Evaluation of Central Blood Pressure in an Asian Population: Comparison between Brachial Oscillometry and Radial Tonometry Methods. Pulse.

[34] Weber T., Wassertheurer S., Rammer M., Maurer E., Hametner B., Mayer C.C., Kropf J., Eber B. (2011). Validation of a Brachial Cuff-Based Method for Estimating Central Systolic Blood Pressure. Hypertension.

[35] Weiss W., Gohlisch C., Harsch-Gladisch C., Tölle M., Zidek W., van der Giet M. (2012). Oscillometric estimation of central blood pressure: Validation of the Mobil-O-Graph in comparison with the SphygmoCor device. Blood Press. Monit..

[36] Wassertheurer S., Kropf J., Weber T., Van Der Giet M., Baulmann J., Ammer M., Hametner B., Mayer C.C., Eber B., Magometschnigg D. (2010). A new oscillometric method for pulse wave analysis: Comparison with a common tonometric method. J. Hum. Hypertens..

[37] D’Agostino R.B., Vasan R.S., Pencina M.J., Wolf P.A., Cobain M., Massaro J.M., Kannel W.B. (2008). General Cardiovascular Risk Profile for Use in Primary Care: The Framingham Heart Study. Circulation.

[38] Hosmer D., Lemeshow S. (2000). Applied Logistic Regression.

[39] Ben-Shlomo Y., Spears M., Boustred C., May M., Anderson S., Benjamin E., Boutouyrie P., Cameron J., Chen C.-H., Cruickshank J.K. (2014). Aortic Pulse Wave Velocity Improves Cardiovascular Event Prediction. J. Am. Coll. Cardiol..

[40] Ji C., Gao J., Huang Z., Chen S., Wang G., Wu S., Jonas J.B. (2020). Estimated pulse wave velocity and cardiovascular events in Chinese. Int. J. Cardiol. Hypertens..

[41] Padilla J., Fadel P.J. (2017). Prolonged sitting leg vasculopathy: Contributing factors and clinical implications. Am. J. Physiol. Circ. Physiol..

[42] Fleenor B.S., Berrones A.J. (2015). Overview of Arterial Stiffness. Arterial Stiffness Implications and Interventions.

[43] E Spieker L., Lüscher T.F., Noll G. (2003). ETA Receptors Mediate Vasoconstriction of Large Conduit Arteries During Reduced Flow in Humans. J. Cardiovasc. Pharmacol..

[44] Dempsey P.C., Larsen R.N., Dunstan D.W., Owen N., Kingwell B.A. (2018). Sitting Less and Moving More: Implications for hypertension. Hypertension.

[45] Germano-Soares A.H., Andrade-Lima A.H., Menêses A.L., Correia M.A., Parmenter B.J., Tassitano R.M., Cucato G.G., Ritti-Dias R.M. (2018). Association of time spent in physical activities and sedentary behaviors with carotid-femoral pulse wave velocity: A systematic review and meta-analysis. Atherosclerosis.

[46] Cavero-Redondo I., Tudor-Locke C., Álvarez-Bueno C., Cunha P.G., Aguiar E.J., Martínez-Vizcaíno V. (2019). Steps per Day and Arterial Stiffness. Hypertension.

[47] Veronese N., Sigeirsdottir K., Eiriksdottir G., Marques E., Chalhoub D., Phillips C.L., Launer L.J., Maggi S., Gudnason V., Harris T.B. (2017). Frailty and Risk of Cardiovascular Diseases in Older Persons: The Age, Gene/Environment Susceptibility-Reykjavik Study. Rejuvenation Res..

[48] Correll C.U., Solmi M., Veronese N., Bortolato B., Rosson S., Santonastaso P., Thapa-Chhetri N., Fornaro M., Gallicchio D., Collantoni E. (2017). Prevalence, incidence and mortality from cardiovascular disease in patients with pooled and specific severe mental illness: A large-scale meta-analysis of 3,211,768 patients and 113,383,368 controls. World Psychiatry.

[49] Huang Q.-F., Aparicio L.S., Thijs L., Wei F.-F., Melgarejo J.D., Cheng Y.-B., Sheng C.-S., Yang W.-Y., Gilis-Malinowska N., Boggia J. (2020). Cardiovascular End Points and Mortality Are Not Closer Associated with Central than Peripheral Pulsatile Blood Pressure Components. Hypertension.

[50] Li W.-F., Huang Y.-Q., Feng Y.-Q. (2019). Association between central haemodynamics and risk of all-cause mortality and cardiovascular disease: A systematic review and meta-analysis. J. Hum. Hypertens..

[51] Lamarche F., Agharazii M., Madore F., Goupil R. (2021). Prediction of Cardiovascular Events by Type I Central Systolic Blood Pressure: A Prospective Study. Hypertension.

[52] Zheng J., Sun Z., Guo X., Xie Y., Sun Y., Zheng L. (2019). Blood pressure predictors of stroke in rural Chinese dwellers with hypertension: A large-scale prospective cohort study. BMC Cardiovasc. Disord..

[53] Lv Y.-B., Gao X., Yin Z.-X., Chen H.-S., Luo J.-S., Brasher M.S., Kraus V.B., Li T.-T., Zeng Y., Shi X.-M. (2018). Revisiting the association of blood pressure with mortality in oldest old people in China: Community based, longitudinal prospective study. BMJ.

[54] Anker D., Santos-Eggimann B., Zwahlen M., Santschi V., Rodondi N., Wolfson C., Chiolero A. (2019). Blood pressure in relation to frailty in older adults: A population-based study. J. Clin. Hypertens..

[55] Aprahamian I., Sassaki E., Dos Santos M.F., Izbicki R., Pulgrossi R.C., Biella M.M., Borges A.C.N., Sassaki M.M., Torres L.M., Fernandez S. (2018). Hypertension and frailty in older adults. J. Clin. Hypertens..

[56] Bastos-Barbosa R.G., Ferriolli E., Coelho E.B., Moriguti J.C., Nobre F., Lima N.K.D.C. (2012). Association of Frailty Syndrome in the Elderly with Higher Blood Pressure and Other Cardiovascular Risk Factors. Am. J. Hypertens..

[57] Coelho-Junior H.J., Uchida M.C., Picca A., Calvani R., Landi F., Gonçalves I.D.O., Rodrigues B., Bernabei R., Marzetti E. (2021). Frailty is not associated with hypertension, blood pressure or antihypertensive medication in community-dwelling older adults: A cross-sectional comparison across 3 frailty instruments. Exp. Gerontol..

[58] Fattori A., Santimaria M., Alves R., Guariento M., Neri A. (2013). Influence of blood pressure profile on frailty phenotype in community-dwelling elders in Brazil—FIBRA study. Arch. Gerontol. Geriatr..

[59] Gijón-Conde T., Graciani A., López-García E., García-Esquinas E., Laclaustra M., Ruilope L.M., Rodríguez-Artalejo F., Banegas J.R. (2018). Frailty, Disability, and Ambulatory Blood Pressure in Older Adults. J. Am. Med. Dir. Assoc..

[60] Ricci N., Pessoa G.S., Ferrioli E., Dias R.C., Perracini M.R. (2014). Frailty and cardiovascular risk in community-dwelling elderly: A population-based study. Clin. Interv. Aging.

[61] Basile G., Catalano A., Mandraffino G., Maltese G., Alibrandi A., Ciancio G., Lasco A., Cesari M. (2017). Relationship between blood pressure and frailty in older hypertensive outpatients. Aging Clin. Exp. Res..

[62] Kang M.-G., Kim S.-W., Yoon S.-J., Choi J.-Y., Kim K.-I., Kim C.-H. (2017). Association between Frailty and Hypertension Prevalence, Treatment, and Control in the Elderly Korean Population. Sci. Rep..

[63] Rockwood M.R., Howlett S.E. (2011). Blood Pressure in Relation to Age and Frailty. Can. Geriatr. J..

[64] Ashor A.W., Lara J., Siervo M., Celis-Morales C., Mathers J.C. (2014). Effects of Exercise Modalities on Arterial Stiffness and Wave Reflection: A Systematic Review and Meta-Analysis of Randomized Controlled Trials. Li, Y., Ed. PLoS ONE.

[65] Cornelissen V.A., Smart N.A. (2013). Exercise training for blood pressure: A systematic review and meta-analysis. J. Am. Heart Assoc..

[66] Tanaka H. (2019). Antiaging Effects of Aerobic Exercise on Systemic Arteries. Hypertension.

[67] Ceciliato J., Costa E.C., Azevêdo L., Sousa J.C., Fecchio R.Y., Brito L.C. (2020). Effect of Resistance Training on Arterial Stiffness in Healthy Subjects: A Systematic Review and Meta-Analysis. Curr. Hypertens. Rep..

