# Supplementary material for: Pre-Frailty Phenotype and Arterial Stiffness in Older Adults Free of Cardiovascular Diseases
Source: Int J Environ Res Public Health. 2022 Oct 18;19(20):13469. doi: 10.3390/ijerph192013469 (PMC9603482; doi:10.3390/ijerph192013469)
Supplement: Supplementary file 1 [file ijerph-19-13469-s001.zip › Table S1.pdf]

Table S1. Characteristics of the participants based on the original Fried criteria

	**Overall**	**Robust**	**Pre-frail**	**P-value**
n (%)	259	102 (39.4)	157 (60.6)	
Age, years	66.0 ± 5.3	66.4 ± 5.5	65.8 ± 5.3	0.342
Race, n (%)				
Caucasian	96 (37.1)	34 (33.3)	62 (39.5)	0.746
Brown	139 (53.7)	59 (57.8)	80 (51.0)	
Black	19 (7.3)	7 (6.9)	12 (7.6)	
Other	5 (1.9)	2 (2.0)	3 (1.9)	
Females, n (%)	206 (79.5)	81 (79.4)	125 (79.6)	0.968
Living with partner, n (%)	166 (64.1)	75 (73.5)	91 (58.0)	**0.011**
Post-secondary education, n (%)	52 (20.1)	19 (18.6)	33 (21.0)	0.639
Body mass index, kg/m^2	28.9 ± 4.6	28.2 ± 4.2	29.4 ± 4.8	**0.030**
Fasting glucose, mg/dL	110.7 ± 31.9	112.8 ± 36.5	109.3 ± 28.6	0.391
Triglycerides, md/dL	154 ± 75.1	154.8 ± 93.5	153.4 ± 60.4	0.881
HDL-cholesterol, md/dL	45.9 ± 12.7	45.7 ± 12.8	46.1 ± 12.7	0.837
LDL-cholesterol, md/dL	132.7 ± 44.3	131.2 ± 43.5	133.7 ± 45	0.652
Total cholesterol, md/dL	205.4 ± 46.6	202.1 ± 43.3	207.6 ± 48.7	0.359
Antihypertensive medication, n (%)				
Monotherapy	74 (47.7)	29 (50.9)	45 (45.9)	0.551
Combination therapy	81 (52.3)	28 (49.1)	53 (54.1)	0.551
Calcium channel blockers	15 (9.7)	4 (7)	11 (11.2)	0.393
Diuretics	63 (40.6)	19 (33.3)	44 (44.9)	0.158
Angiotensin II receptor blockers	118 (76.1)	41 (71.9)	77 (78.6)	0.350
ACE inhibitors	15 (9.7)	7 (12.3)	8 (8.2)	0.403

Beta-blockers	39 (25.2)	19 (33.3)	20 (20.4)	0.074
Diabetes medication, n (%)	67 (25.9)	28 (27.5)	39 (24.8)	0.639
Lipid medication, n (%)	84 (32.4)	28 (27.5)	56 (35.7)	0.167
Ex-smoker/smoker, n (%)	113 (43.6)	43 (42.2)	70 (44.6)	0.700
Framingham risk, n (%)				
Low risk	87 (34.1)	39 (38.6)	48 (31.2)	0.464
Moderate risk	126 (49.4)	46 (45.5)	80 (51.9)	
High risk	42 (16.5)	16 (15.8)	26 (16.9)	
Central SBP, mmHg	120.8 ± 16.3	117.9 ± 16.3	122.7 ± 16.1	**0.019**
Central DBP, mmHg	82.0 ± 11.1	80.4 ± 10.9	83.0 ± 11.2	0.058
Central MBP, mmHg	94.9 ± 12.3	92.8 ± 12.0	96.3 ± 12.3	**0.029**
Central PP, mmHg	38.8 ± 9.5	37.5 ± 10.1	39.7 ± 9.0	0.071
Brachial SBP, mmHg	128.2 ± 17.4	124.6 ± 17.2	130.5 ± 17.1	**0.008**
Brachial DBP, mmHg	80.9 ± 10.9	79.2 ± 10.6	82.0 ± 11.0	**0.039**
Brachial MBP, mmHg	96.7 ± 12.4	94.3 ± 12.0	98.2 ± 12.4	**0.014**
Brachial PP, mmHg	47.3 ± 11.0	45.5 ± 11.5	48.5 ± 12.4	**0.032**
Aortic pulse wave velocity, m/s	9.5 ± 1.1	9.5 ± 1.1	9.6 ± 1.0	0.569
Fried frailty, n (%)				
Low physical activity	-	-	97 (37.4)	
Exhaustion	-	-	51 (19.7)	
Weakness	-	-	34 (13.1)	
Unintentional weight loss	-	-	31 (12.0)	
Slowness	-	-	01 (0.4)	

Values are shown as mean ± SD or absolute (n) and relative (%) frequency.

Bold values indicate statistical significance ($p < 0.05$).

Abbreviations: ACE, angiotensin-converting-enzyme; BP, blood pressure; DBP, diastolic blood pressure; HDL, high-density lipoprotein; LDL, low-density lipoprotein; SBP, systolic blood pressure; MBP, mean blood pressure; PP, pulse pressure.
