# Supplementary material for: Pre-Frailty Phenotype and Arterial Stiffness in Older Adults Free of Cardiovascular Diseases
Source: Int J Environ Res Public Health. 2022 Oct 18;19(20):13469. doi: 10.3390/ijerph192013469 (PMC9603482; doi:10.3390/ijerph192013469)
Supplement: Supplementary file 1 [file ijerph-19-13469-s001.zip › Table S2.pdf]

Table S2. Coefficient estimates for aortic pulse wave velocity and blood pressure (central and brachial) among pre-frail vs robust older adults based on the standardized Fried criteria

	**Age-adjusted model**			**Full-adjusted model[a]**		
	**β**	**95% CI**	**P-value**	**β**	**95% CI**	**P-value**
Aortic pulse wave velocity, m/s	0.19	0.05, 0.34	**0.008**	0.19	0.06, 0.35	**0.007**
Central SBP, mmHg	4.7	0.6, 8.8	**0.025**	4.8	0.7, 8.9	**0.021**
Central DBP, mmHg	1.9	-0.8, 4.7	0.168	2.0	-0.7, 4.8	0.151
Central MBP, mmHg	2.8	-0.2, 5.9	0.067	3.0	-0.1, 6.1	0.059
Central PP, mmHg	2.8	0.3, 5.2	**0.025**	2.9	0.6, 5.1	**0.014**
Brachial SBP, mmHg	5.2	0.8, 9.5	**0.020**	5.3	1.0, 9.7	**0.017**
Brachial DBP, mmHg	2.0	-0.7, 4.7	0.139	2.1	-0.6, 4.8	0.129
Brachial MBP, mmHg	3.1	0.0, 6.1	0.050	3.1	0.0, 6.2	**0.046**
Brachial PP, mmHg	3.1	0.3, 5.8	**0.029**	3.3	0.6, 5.9	**0.017**

Values are shown as coefficient estimates (β) and 95% confidence interval (CI).

[a]Adjusted model for age, sex, body mass index categories, post-secondary education and hypertension medication.

Bold values indicate statistical significance ($p < 0.05$).

Abbreviations: BP, blood pressure; SBP, systolic blood pressure; DBP, diastolic blood pressure; MBP, mean blood pressure; PP, pulse pressure.
