# Supplementary material for: Pre-Frailty Phenotype and Arterial Stiffness in Older Adults Free of Cardiovascular Diseases
Source: Int J Environ Res Public Health. 2022 Oct 18;19(20):13469. doi: 10.3390/ijerph192013469 (PMC9603482; doi:10.3390/ijerph192013469)
Supplement: Supplementary file 1 [file ijerph-19-13469-s001.zip › Table S3.pdf]

Table S3. Coefficient estimates for aortic pulse wave velocity and blood pressure (central and brachial) among pre-frail vs robust older adults based on the original Fried criteria

	**Age-adjusted model**			**Full-adjusted model[a]**		
	**β**	**95% CI**	**P-value**	**β**	**95% CI**	**P-value**
Aortic pulse wave velocity, m/s	0.19	0.05, 0.33	**0.008**	0.19	0.05, 0.33	**0.010**
Central SBP, mmHg	4.9	0.9, 8.9	**0.017**	4.7	0.6, 8.8	**0.023**
Central DBP, mmHg	2.6	-0.1, 5.4	0.064	2.4	-0.4, 5.2	0.090
Central MBP, mmHg	3.4	0.4, 6.4	**0.027**	3.2	0.1, 6.3	**0.043**
Central PP, mmHg	2.3	-0.1, 4.7	0.058	2.3	0.05, 4.58	**0.045**
Brachial SBP, mmHg	6.0	1.7, 10.2	**0.006**	5.8	1.6, 10.1	**0.007**
Brachial DBP, mmHg	2.8	0.1, 5.5	**0.044**	2.6	-0.1, 5.4	0.058
Brachial MBP, mmHg	3.8	0.8, 6.9	**0.013**	3.7	0.6, 6.8	**0.018**
Brachial PP, mmHg	3.2	0.5, 5.9	**0.020**	3.2	0.6, 5.8	**0.016**

Values are shown as coefficient estimates (β) and 95% confidence interval (CI).

[a]Adjusted model for age, sex, body mass index categories, post-secondary education and hypertension medication.

Bold values indicate statistical significance ($p < 0.05$).

Abbreviations: BP, blood pressure; SBP, systolic blood pressure; DBP, diastolic blood pressure; MBP, mean blood pressure; PP, pulse pressure.
