# Supplementary material for: Pre-Frailty Phenotype and Arterial Stiffness in Older Adults Free of Cardiovascular Diseases
Source: Int J Environ Res Public Health. 2022 Oct 18;19(20):13469. doi: 10.3390/ijerph192013469 (PMC9603482; doi:10.3390/ijerph192013469)
Supplement: Supplementary file 1 [file ijerph-19-13469-s001.zip › Table S4.pdf]

Table S4. Characteristics of the participants based on the standardized Fried criteria according to sex

	Overall		Robust		Pre-frail		
	**Males**	**Females**	**Males**	**Females**	**Males**	**Females**	**P-value**
N (%)	51	198	21 (41.2)	74 (37.4)	30 (58.8)	124 (62.6)	
Age, years	67.3 ± 5.5	65.8 ± 5.3	68.3 ± 6	66 ± 5	66.6 ± 5.1	65.8 ± 5.4	0.278
Race, n (%)							
Caucasian	17 (33.3)	72 (36.4)	4 (19)	28 (37.8)	13 (43.3)	44 (35.5)	0.301
Brown	29 (56.9)	109 (55.1)	15 (71.4)	41 (55.4)	14 (46.7)	68 (54.8)	
Black	3 (5.9)	14 (7.1)	1 (4.8)	5 (6.8)	2 (6.7)	9 (7.3)	
Other	2 (3.9)	3 (1.5)	1 (4.8)	0 (0)	1 (3.3)	3 (2.4)	
Living with partner, n (%)	45 (88.2)	114 (57.6)	18 (85.7)	50 (67.6)	27 (90)	64 (51.6)	**0.028***
Post-secondary education, n (%)	12 (23.5)	39 (19.7)	2 (9.5)	14 (18.9)	10 (33.3)	25 (20.2)	**0.049**[†]
Body mass index, kg/m^2	28.2 ± 4.1	29.2 ± 4.7	27.4 ± 3.8	29 ± 4.4	28.8 ± 4.2	29.3 ± 4.9	0.209
Fasting glucose, mg/dL	117.3 ± 39.8	109.6 ± 29.5	123.9 ± 55	108.2 ± 29.4	112.5 ± 23.6	110.4 ± 29.7	0.323

Triglycerides, md/dL	159.6 ± 77	153 ± 74.6	158 ± 91.7	157.9 ± 90	160.7 ± 66	150.1 ± 63.9	0.477
HDL-cholesterol, md/dL	40.4 ± 11.1	47.4 ± 12.7	40 ± 9.7	46.9 ± 11.7	40.8 ± 12.2	47.6 ± 13.3	0.693
LDL-cholesterol, md/dL	123.3 ± 44.1	134.7 ± 44.5	128.2 ± 50.1	136.9 ± 46.7	119.7 ± 39.8	133.4 ± 43.3	0.503
Total cholesterol, md/dL	189.7 ± 44	209.1 ± 46.6	193.7 ± 49.8	209.4 ± 47.3	186.8 ± 40	208.8 ± 46.4	0.590
Antihypertensive medication, n (%)							
Monotherapy	19 (57.6)	54 (45.4)	7 (58.3)	23 (48.9)	12 (57.1)	31 (43.1)	0.529
Combination therapy	14 (42.4)	65 (54.6)	5 (41.7)	24 (51.1)	9 (42.9)	41 (56.9)	0.529
Calcium channel blockers	5 (15.2)	11 (9.2)	2 (16.7)	4 (8.5)	3 (14.3)	7 (9.7)	0.823
Diuretics	10 (30.3)	51 (42.9)	3 (25)	16 (34)	7 (33.3)	35 (48.6)	0.116
Angiotensin II receptor blockers	25 (75.8)	91 (76.5)	9 (75)	36 (76.6)	16 (76.2)	55 (76.4)	0.939
ACE inhibitors	2 (6.1)	12 (10.1)	1 (8.3)	4 (8.5)	1 (4.8)	8 (11.1)	0.645
Beta-blockers	7 (21.2)	32 (26.9)	3 (25)	17 (36.2)	4 (19)	15 (20.8)	0.065
Diabetes medication, n (%)	14 (27.5)	50 (25.3)	6 (28.6)	23 (31.1)	8 (26.7)	27 (21.8)	0.145
Lipid medication, n (%)	18 (35.3)	62 (31.3)	4 (19)	20 (27)	14 (46.7)	42 (33.9)	**0.042**†

Ex-smoker/smoker, n (%)	31 (60.8)	79 (39.9)	13 (61.9)	27 (36.5)	18 (60)	52 (41.9)	0.449
Framingham risk, n (%)							
Low risk	21 (42)	61 (31.1)	10 (47.6)	26 (35.6)	11 (37.9)	35 (28.5)	0.428
Moderate risk	29 (58)	92 (46.9)	11 (52.4)	30 (41.1)	18 (62.1)	62 (50.4)	
High risk	-	43 (21.9)	-	17 (23.3)	-	26 (21.1)	
Central SBP, mmHg	121.5 ± 16.7	120.8 ± 16.3	117.4 ± 12.9	118.3 ± 16.6	124.4 ± 18.6	122.3 ± 16	0.091
Central DBP, mmHg	86.3 ± 11.8	81.1 ± 11	83.9 ± 8.5	80 ± 9.8	88 ± 13.5	81.7 ± 11.7	0.224
Central MBP, mmHg	98 ± 13	94.3 ± 12.2	95 ± 9.6	92.8 ± 11.5	100.1 ± 14.7	95.2 ± 12.6	0.172
Central PP, mmHg	35.2 ± 8.7	39.8 ± 9.5	33.5 ± 7.5	38.2 ± 10	36.4 ± 9.3	40.7 ± 9	0.082
Brachial SBP, mmHg	129 ± 17.3	128.2 ± 17.5	124.5 ± 14.1	125.5 ± 17.5	132.1 ± 18.7	129.8 ± 17.4	0.090
Brachial DBP, mmHg	84.9 ± 11.4	80 ± 10.9	82.5 ± 8.1	78.9 ± 9.6	86.5 ± 13.1	80.7 ± 11.5	0.220
Brachial MBP, mmHg	99.6 ± 12.9	96.1 ± 12.3	96.5 ± 9.6	94.4 ± 11.5	101.7 ± 14.6	97.1 ± 12.8	0.145
Brachial PP, mmHg	44.1 ± 9.2	48.2 ± 11.3	42 ± 9	46.6 ± 11.9	45.6 ± 9.2	49.1 ± 10.9	0.121
Aortic pulse wave velocity, m/s	9.7 ± 1	9.5 ± 1.1	9.8 ± 1.2	9.4 ± 1.1	9.7 ± 0.9	9.6 ± 1.1	0.4.35

Frailty criteria, n (%)						
Low physical activity	-	-	0	0	11 (36.7)	44 (35.5)
Exhaustion	-	-	0	0	6 (20)	41 (33.1)
Weakness	-	-	0	0	10 (33.3)	31 (25)
Unintentional weight loss	-	-	0	0	6 (20)	23 (18.5)
Slowness	-	-	0	0	5 (16.7)	34 (27.4)

Values are shown as mean ± SD or absolute (n) and relative (%) frequency.

Bold values indicate statistical significance ($p < 0.05$).

*Difference between pre-frail vs. robust phenotype in the female group.

†Difference between pre-frail vs. robust phenotype in the male group.

Abbreviations: ACE, angiotensin-converting-enzyme; BP, blood pressure; DBP, diastolic blood pressure; HDL, high-density lipoprotein; LDL, low-density lipoprotein; SBP, systolic blood pressure; MBP, mean blood pressure; PP, pulse pressure.
