# Supplementary material for: Pre-Frailty Phenotype and Arterial Stiffness in Older Adults Free of Cardiovascular Diseases
Source: Int J Environ Res Public Health. 2022 Oct 18;19(20):13469. doi: 10.3390/ijerph192013469 (PMC9603482; doi:10.3390/ijerph192013469)
Supplement: Supplementary file 1 [file ijerph-19-13469-s001.zip › Table S5.pdf]

Table S5. Characteristics of the participants based on the original Fried criteria according to sex

	Overall		Robust		Pre-frail		
	**Males**	**Females**	**Males**	**Females**	**Males**	**Females**	**P-value**
N (%)	53	206	21 (39.6)	81 (39.3)	32 (60.4)	125 (60.7)	
Age, years	67.3 ± 5.4	65.7 ± 5.3	68.0 ± 6.2	66.0 ± 5.2	66.8 ± 4.8	65.5 ± 5.3	0.422
Race, n (%)							
Caucasian	17 (32.1)	79 (38.3)	05 (23.8)	29 (35.8)	12 (37.5)	50 (40.0)	0.272
Brown	31 (58.5)	108 (52.4)	13 (61.9)	46 (56.8)	18 (56.3)	62 (49.6)	
Black	03 (5.7)	16 (7.8)	01 (4.8)	06 (7.4)	02 (6.3)	10 (8.0)	
Other	02 (3.8)	03 (1.5)	02 (9.5)	-	-	03 (2.4)	
Living with partner, n (%)	46 (86.8)	120 (58.3)	19 (90.5)	56 (69.1)	27 (84.4)	64 (51.2)	**0.011***
Post-secondary education, n (%)	12 (22.6)	40 (19.4)	04 (19.0)	15 (18.5)	08 (25.0)	25 (20.0)	0.613
Body mass index, kg/m^2	28.2 ± 4.0	29.1 ± 4.7	27.3 ± 3.8	28.4 ± 4.2	28.7 ± 4.1	29.6 ± 4.9	0.069
Fasting glucose, mg/dL	116.7 ± 39.6	109.2 ± 29.7	124.6 ± 54.6	109.7 ± 29.8	111.2 ± 24	108.8 ± 29.7	0.240
Triglycerides, md/dL	160.2 ± 76.4	152.4 ± 74.9	149.8 ± 91.7	156.1 ± 94.5	167.6 ± 64.2	150 ± 59.2	0.418
HDL-cholesterol, md/dL	40.1 ± 11.3	47.4 ± 12.6	41.3 ± 11.5	46.9 ± 12.9	39.2 ± 11.3	47.7 ± 12.5	0.522
LDL-cholesterol, md/dL	123.6 ± 43.8	135 ± 44.3	129.4 ± 46.2	131.6 ± 43	119.6 ± 42.3	137.2 ± 45.1	0.386

Total cholesterol, md/dL	189.9 ± 43.6	209.3 ± 46.6	195 ± 42.8	204 ± 43.4	186.3 ± 44.5	2012.7 ± 48.5	0.190
Antihypertensive medication, n (%)							
Monotherapy	19 (57.9)	55 (45.1)	6 (54.5)	23 (50)	13 (59.1)	32 (42.1)	0.396
Combination therapy	14 (42.4)	67 (54.9)	5 (45.5)	23 (50)	9 (40.9)	44 (57.9)	0.396
Calcium channel blockers	5 (15.2)	10 (8.2)	2 (18.2)	2 (4.3)	3 (13.6)	8 (10.5)	0.228
Diuretics	10 (30.3)	53 (43.4)	3 (27.3)	16 (34.8)	7 (31.8)	37 (48.7)	0.133
Angiotensin II receptor blockers	25 (75.8)	93 (76.2)	8 (72.7)	33 (71.7)	17 (77.3)	60 (78.9)	0.365
ACE inhibitors	2 (6.1)	13 (10.7)	1 (9.1)	6 (13)	1 (4.5)	7 (9.2)	0.506
Beta-blockers	7 (21.2)	32 (26.2)	3 (27.3)	16 (34.8)	4 (18.2)	16 (21.1)	0.095
Diabetes medication, n (%)	14 (26.4)	53 (25.7)	05 (23.8)	23 (28.4)	09 (28.1)	30 (24.0)	0.481
Lipid medication, n (%)	18 (34.0)	66 (32.0)	04 (19.0)	24 (29.6)	14 (43.8)	42 (33.6)	0.063
Ex-smoker/smoker, n (%)	33 (62.3)	80 (38.8)	13 (61.9)	30 (37.0)	20 (62.5)	50 (40.0)	0.670
Framingham risk, n (%)							
Low risk	21 (41.2)	66 (32.4)	11 (52.4)	28 (35.0)	10 (33.3)	38 (30.6)	0.174
Moderate risk	30 (58.8)	96 (47.1)	10 (47.6)	36 (45.0)	20 (66.7)	60 (48.4)	
High risk	-	42 (20.6)	-	16 (20.0)	-	26 (21.0)	
Central SBP, mmHg	121.6 ± 17.0	120.6 ± 16.1	116.5 ± 13.5	118.2 ± 16.9	125.0 ± 18.4	122.2 ± 15.5	0.077

Central DBP, mmHg	86.4 ± 11.8	80.8 ± 10.7	84.1 ± 9.2	79.4 ± 11.1	87.9 ± 13.2	81.8 ± 10.4	0.112
Central MBP, mmHg	98.1 ± 13.2	94.1 ± 12.0	94.9 ± 10.2	92.3 ± 12.4	100.2 ± 14.5	95.2 ± 11.6	0.086
Central PP, mmHg	35.2 ± 8.8	39.8 ± 10.3	32.4 ± 7.5	38.9 ± 10.3	37.1 ± 9.1	40.4 ± 8.9	0.054
Brachial SBP, mmHg	129.1 ± 17.8	128.0 ± 17.3	123.6 ± 15.0	124.9 ± 17.8	132.7 ± 18.8	130.0 ± 16.7	**0.040***
Brachial DBP, mmHg	85.0 ± 11.4	79.8 ± 10.6	82.8 ± 8.8	78.2 ± 10.9	86.4 ± 12.8	80.9 ± 10.2	0.073
Brachial MBP, mmHg	99.7 ± 13.1	95.9 ±12.1	96.4 ± 10.3	93.8 ± 12.4	101.8 ± 14.4	97.3 ± 11.7	**0.043***
Brachial PP, mmHg	44.1 ± 9.6	48.1 ± 11.2	40.8 ± 9.4	46.7 ± 11.8	46.3 ± 9.3	49.1 ± 10.8	**0.042**†
Aortic pulse wave velocity, m/s	9.8 ± 1.0	9.5 ± 1.1	9.7 ± 1.3	9.4 ± 1.1	9.8 ± 0.8	9.5 ± 1.1	0.627
Frailty criteria, n (%)							
Low physical activity	-	-	0	0	19 (35.8)	78 (37.9)	
Exhaustion	-	-	0	0	08 (15.1)	43 (20.9)	
Weakness	-	-	0	0	07 (13.2)	27 (13.1)	
Unintentional weight loss	-	-	0	0	06 (11.3)	25 (12.1)	
Slowness	-	-	0	0	00 (0.0)	01 (0.5)	

Values are shown as mean ± SD or absolute (n) and relative (%) frequency.

Bold values indicate statistical significance ($p < 0.05$).

*Difference between pre-frail vs. robust phenotype in the female group.

†Difference between pre-frail vs. robust phenotype in the male group.

Abbreviations: ACE, angiotensin-converting-enzyme; BP, blood pressure; DBP, diastolic blood pressure; HDL, high-density lipoprotein; LDL, low-density lipoprotein; SBP, systolic blood pressure; MBP, mean blood pressure; PP, pulse pressure.
