# Supplementary material for: Pre-Frailty Phenotype and Arterial Stiffness in Older Adults Free of Cardiovascular Diseases
Source: Int J Environ Res Public Health. 2022 Oct 18;19(20):13469. doi: 10.3390/ijerph192013469 (PMC9603482; doi:10.3390/ijerph192013469)
Supplement: Supplementary file 1 [file ijerph-19-13469-s001.zip › Table S6.pdf]

Table S6. Coefficient estimates for aortic pulse wave velocity and blood pressure (central and brachial) among pre-frail vs. robust older adults based on the standardized Fried criteria according to sex

	**Age-adjusted model**						**Full-adjusted model[a]**					
	**Males (n = 51)**			**Females (n = 198)**			**Males (n = 51)**			**Females (n = 198)**		
	**β**	**95% CI**	**P-value**	**β**	**95% CI**	**P-value**	**β**	**95% CI**	**P-value**	**β**	**95% CI**	**P-value**
Aortic pulse wave velocity, m/s	0.28	-0.01, 0.58	0.059	0.17	0.01, 0.33	**0.037**	0.32	0.04, 0.59	**0.023**	0.18	0.01, 0.35	**0.043**
Central SBP, mmHg	6.3	-2.7, 15.3	0.168	4.1	-0.5, 8.6	0.078	9.0	0.0, 18.1	**0.050**	4.3	-0.2, 8.8	0.059
Central DBP, mmHg	3.1	-3.3, 9.5	0.341	1.6	-1.5, 4.7	0.319	5.0	-0.8, 10.8	0.089	1.8	-1.3, 4.9	0.254
Central MBP, mmHg	4.4	-2.2, 11.0	0.190	2.4	-1.0, 5.9	0.164	6.4	0.0, 12.8	**0.049**	2.7	-0.9, 6.2	0.137
Central PP, mmHg	3.1	-1.4, 7.5	0.176	2.5	-0.3, 5.2	0.075	4.9	0.2, 9.6	**0.042**	2.4	-0.1, 5.0	0.060
Brachial SBP, mmHg	7.2	-2.1, 16.5	0.127	4.4	-0.4, 9.3	0.073	9.6	0.2, 19.0	**0.045**	4.8	-0.1, 9.6	0.053
Brachial DBP, mmHg	3.0	-3.2, 9.2	0.339	1.7	-1.3, 4.8	0.270	4.7	-0.8, 10.3	0.093	1.9	-1.1, 5.0	0.217
Brachial MBP, mmHg	4.4	-2.6, 11.4	0.214	2.6	-0.8, 6.1	0.137	6.4	0.1, 12.7	**0.046**	2.9	-0.7, 6.5	0.113
Brachial PP, mmHg	4.0	-0.8, 8.9	0.104	2.7	-0.5, 5.8	0.097	5.1	0.6, 9.5	**0.027**	2.9	-0.4, 6.1	0.081

Values are shown as coefficient estimates (β) and 95% confidence interval (CI).

[a]Adjusted model for age, body mass index categories, post-secondary education and hypertension medication.

Bold values indicate statistical significance ($p < 0.05$).

Abbreviations: BP, blood pressure; SBP, systolic blood pressure; DBP, diastolic blood pressure; MBP, mean blood pressure; PP, pulse pressure.
