# Supplementary material for: Pre-Frailty Phenotype and Arterial Stiffness in Older Adults Free of Cardiovascular Diseases
Source: Int J Environ Res Public Health. 2022 Oct 18;19(20):13469. doi: 10.3390/ijerph192013469 (PMC9603482; doi:10.3390/ijerph192013469)
Supplement: Supplementary file 1 [file ijerph-19-13469-s001.zip › Table S7.pdf]

Table S7. Coefficient estimates for aortic pulse wave velocity and blood pressure (central and brachial) among pre-frail vs. robust older adults based on the original Fried criteria according to sex

	**Age-adjusted model**						**Full-adjusted model**[a]					
	**Males (n = 53)**			**Females (n = 206)**			**Males (n = 53)**			**Females (n = 206)**		
	**β**	**95% CI**	**P-value**	**β**	**95% CI**	**P-value**	**β**	**95% CI**	**P-value**	**β**	**95% CI**	**P-value**
Aortic pulse wave velocity, m/s	0.35	0.06, 0.63	**0.018**	0.17	0.00, 0.33	**0.039**	0.37	0.07, 0.66	**0.014**	0.16	-0.0, 0.3	0.053
Central SBP, mmHg	8.0	-0.6, 16.6	0.067	4.0	-0.5, 8.6	0.082	9.2	0.3, 18.1	**0.043**	3.8	-0.8, 8.4	0.104
Central DBP, mmHg	3.2	-2.9, 9.2	0.302	2.4	-0.6, 5.4	0.123	3.8	-2.7, 10.2	0.252	2.2	-0.9, 5.3	0.160
Central MBP, mmHg	4.5	-2.4, 11.4	0.202	2.9	-0.4, 6.3	0.089	5.6	-1.5, 12.7	0.124	2.7	-0.7, 6.1	0.118
Central PP, mmHg	5.0	0.7, 9.4	**0.024**	1.7	-1.0, 4.3	0.219	5.7	1.8, 9.6	**0.004**	1.6	-1.0, 4.2	0.240
Brachial SBP, mmHg	8.7	-0.4, 17.8	0.061	5.2	0.4, 10.0	**0.034**	9.8	0.3, 19.3	**0.044**	4.9	0.1, 9.7	**0.047**
Brachial DBP, mmHg	3.0	-2.8, 8.8	0.307	2.6	-0.3, 5.6	0.083	3.5	-2.7, 9.6	0.271	2.5	-0.5, 5.5	0.105
Brachial MBP, mmHg	4.9	-1.8, 11.6	0.149	3.5	0.1, 6.9	**0.044**	5.6	-1.5, 12.7	0.125	3.3	-0.1, 6.7	0.060
Brachial PP, mmHg	5.9	0.9, 11.0	**0.022**	2.7	-0.4, 5.7	0.084	6.3	1.7, 11.0	**0.008**	2.4	-0.6, 5.4	0.122

Notes: Values are shown as coefficient estimates (β) and 95% confidence interval (CI).

[a]Adjusted model for age, body mass index categories, post-secondary education and hypertension medication.

Bold values indicate statistical significance ($p < 0.05$).

Abbreviations: BP, blood pressure; SBP, systolic blood pressure; DBP, diastolic blood pressure; MBP, mean blood pressure; PP, pulse pressure.
