# Supplementary figures and images for: Pre-Frailty Phenotype and Arterial Stiffness in Older Adults Free of Cardiovascular Diseases
Source: Int J Environ Res Public Health. 2022 Oct 18;19(20):13469. doi: 10.3390/ijerph192013469 (PMC9603482; doi:10.3390/ijerph192013469)

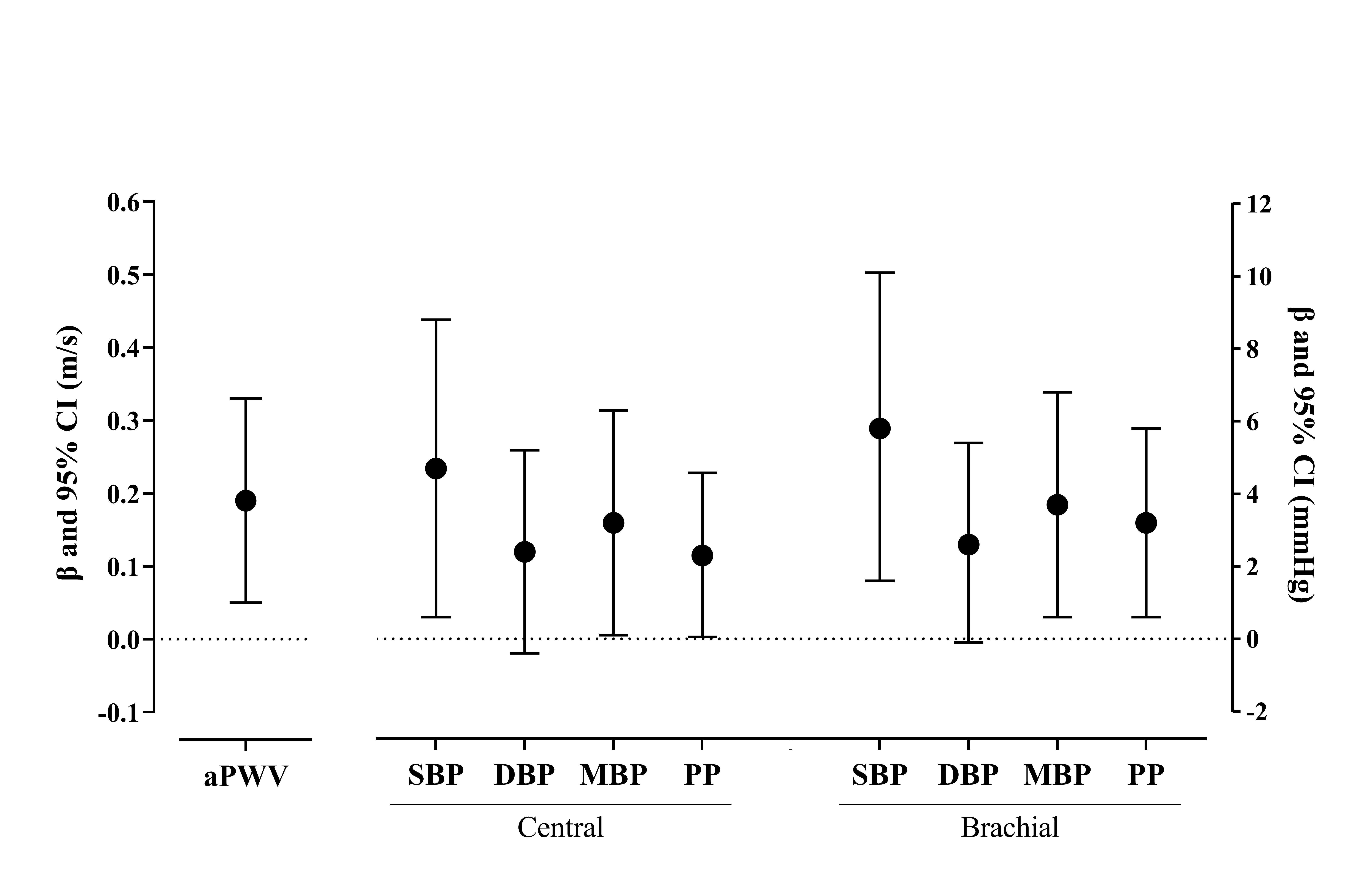

Supplement: Supplementary file 1 [file ijerph-19-13469-s001.zip › Figure S1.tif]
